# *Salmonella enterica* Serovar Typhimurium 14028s Genomic Regions Required for Colonization of Lettuce Leaves

**DOI:** 10.3389/fmicb.2020.00006

**Published:** 2020-01-24

**Authors:** Jeanine Montano, Gabrielle Rossidivito, Joseph Torreano, Steffen Porwollik, Shlomo Sela Saldinger, Michael McClelland, Maeli Melotto

**Affiliations:** ^1^Department of Plant Sciences, University of California, Davis, Davis, CA, United States; ^2^Plant Pathology Graduate Group, University of California, Davis, Davis, CA, United States; ^3^Plant Biology Graduate Group, University of California, Davis, Davis, CA, United States; ^4^Department of Microbiology and Molecular Genetics, University of California, Irvine, Irvine, CA, United States; ^5^Microbial Food Safety Research Unit, Department of Food Science, Agricultural Research Organization, Volcani Center, Rishon LeTsiyon, Israel

**Keywords:** food safety, leafy vegetable, *Salmonella* mutant screen, bacterial persistence, lettuce stress response

## Abstract

Contamination of edible produce leaves with human bacterial pathogens has been associated with serious disease outbreaks and has become a major public health concern affecting all aspects of the market, from farmers to consumers. While pathogen populations residing on the surface of ready-to-eat produce can be potentially removed through thorough washing, there is no disinfection technology available that effectively eliminates internal bacterial populations. By screening 303 multi-gene deletion (MGD) mutants of *Salmonella enterica* serovar Typhimurium (STm) 14028s, we were able to identify ten genomic regions that play a role in opening the stomatal pore of lettuce leaves. The major metabolic functions of the deleted regions are associated with sensing the environment, bacterium movement, transport through the bacterial membrane, and biosynthesis of surface appendages. Interestingly, at 21 days post inoculation, seven of these mutants showed increased population titers inside the leaf, two mutants showed similar titers as the wild type bacterium, whereas one mutant with a large deletion that includes the *Salmonella* pathogenicity island 2 (SPI-2) showed significantly impaired persistence in the leaf apoplast. These findings suggest that not all the genomic regions required for initiation of leaf colonization (i.e., epiphytic behavior and tissue penetration) are essential for continuing bacterial survival as an endophyte. We also observed that mutants lacking either SPI-1 (Mut3) or SPI-2 (Mut9) induce callose deposition levels comparable to those of the wild type STm 14028s; therefore, these islands do not seem to affect this lettuce defense mechanism. However, the growth of Mut9, but not Mut3, was significantly impaired in the leaf apoplastic wash fluid (AWF) suggesting that the STm persistence in the apoplast may be linked to nutrient acquisition capabilities or overall bacterial fitness in this niche, which are dependent on the gene(s) deleted in the Mut9 strain. The genetic basis of STm colonization of leaves investigated in this study provides a foundation from which to develop mitigation tactics to enhance food safety.

## Introduction

Human pathogen contamination of produce was the leading cause of foodborne illnesses and outbreaks associated with a single-ingredient commodity between 2004 and 2013 (Fischer et al., 2015). Lack of visual evidence that indicates the presence of contamination on produce contributes to the estimated 9.4 million cases of foodborne illness in the United States annually (Dewey-Mattia et al., 2016). Various pathogen groups and toxins can be causal agents of foodborne illness associated with produce; however, non-typhoidal *Salmonella* ranks as the second leading cause of all illnesses associated with consumption of produce (DeWaal et al., 2008; Fischer et al., 2015; Dewey-Mattia et al., 2016).

In a pre-harvest setting, enteric pathogenic bacteria are introduced to fresh produce through many routes, including low-quality irrigation water, use of contaminated organic fertilizers, close proximity to livestock operations, wildlife intrusions, improper worker hygiene, or contaminated equipment (Critzer and Doyle, 2010; Barak and Schroeder, 2012). Once on the leaf surface, bacteria are faced with harsh conditions, such as UV irradiation, low nutrient and water availability, and unfavorable weather (Hirano and Upper, 1983; Lindow and Brandl, 2003). Bacteria may escape these conditions by attaching to the leaf surface and forming biofilms (Kroupitski et al., 2009) or by transitioning to an endophytic lifestyle through internalization into the leaf extracellular space (i.e., apoplast) via natural pores or wounds (Kroupitski et al., 2009; Critzer and Doyle, 2010; Roy et al., 2013). While leaf internalization is likely to confer some protection to the bacteria, it is not without some disadvantages. Plants can detect endophytic bacteria in the apoplast through pattern recognition receptors (PRRs) localized at the cell membrane that perceive conserved microbial molecules known as pathogen- or microbe-associated molecular patterns (PAMPs or MAMPs) (Nicaise et al., 2009; Newman et al., 2013). PRR-PAMP binding leads to initiation of PAMP-triggered immunity (PTI) (Jones and Dangl, 2006), which functions to prevent further internalization of bacteria (Melotto et al., 2006; Kroupitski et al., 2009; Roy et al., 2013) and to eradicate those that have already entered the apoplast (Jones and Dangl, 2006; Nicaise et al., 2009). This suggests that internalization trades one challenge for another (i.e., those of the phylloplane for those of the apoplast), and only bacteria that can cope with these challenges will be able to colonize leaves successfully.

Previous studies have shown that *Salmonella* spp. interact with plants in a sophisticated manner, although the exact mechanisms are not fully understood (Melotto et al., 2014). For instance, similar to some plant pathogens, *Salmonella enterica* serovar Typhimurium (STm) SL1344 can modulate stomatal movement in Arabidopsis leaves, where it induces a transient stomatal closure and re-opening at 4 h post incubation (hpi) (Roy et al., 2013). Stomatal closure can diminish bacterial internalization and subsequent contamination of internal leaf tissues. Bacterium-induced re-opening of stomata can lead to higher pathogen load in the leaf apoplast (reviewed by Garcia and Hirt, 2014; Melotto et al., 2014).

The mechanism for stomatal re-opening by the phytopathogen *Pseudomonas syringae* pv. *tomato* (Pst) is through the action of coronatine (Melotto et al., 2006), a polyketide phytotoxin (Bender et al., 1999). However, the genomes of STm strains LT2 and 14028s do not encode genes for coronatine synthesis (McClelland et al., 2001; Jarvik et al., 2010). Furthermore, stomatal re-opening is not a ubiquitous response to human pathogens. For instance, *Escherichia coli* O157:H7 induces a lasting stomatal closure in lettuce and Arabidopsis for at least 4 and 8 h, respectively (Melotto et al., 2006; Roy et al., 2013).

Beyond the ability to modulate stomatal movement, STm SL1344 can survive at a higher titer within the apoplast of Arabidopsis leaves than O157:H7 after surface-inoculation (Roy et al., 2013) and *S. enterica* serovar Thompson strain RM1987 can survive at high titers on the surface of romaine lettuce leaves (Brandl and Amudson, 2008). Therefore, *S. enterica* may either induce a weak plant immune response or can counteract plant immunity and consequently persist on and in leaves (Garcia and Hirt, 2014; Melotto et al., 2014). Internalization and persistence within the apoplast are arguably the most important targets for managing contamination of produce by *Salmonella*, as endophytic populations cannot be removed through typical washing tactics (Heaton and Jones, 2008; Gil et al., 2009; Goodburn and Wallace, 2013).

Here, we provide details of multiple genomic regions required for internalization and persistence of STm 14028s into lettuce (*Lactuca sativa* cv. Salinas) leaves. These genomic regions were identified with a genetic screen of multi-gene deletion (MGD) mutants of STm 14028s (Porwollik et al., 2014) to pinpoint proteins and metabolic pathways responsible for stomatal re-opening and apoplastic persistence. Selected MGD mutants were further characterized regarding their ability to survive in the apoplast, induce hallmark plant defenses, and replicate in apoplastic wash fluid (AWF). While all mutants induced a prolonged stomatal closure when applied to the leaf surface, the mutants were found to vary in other aspects of phyllosphere survival.

## Materials and Methods

### Bacterial Strains and Growth Conditions

*Salmonella enterica* STm 14028s and 303 mutants derived through lambda red-mediated gene recombination (Supplementary Table S1) were obtained from the McClelland laboratory at the University of California, Irvine (Porwollik et al., 2014). The mutants have a MGD that removes 2–70 genes from each strain and the mutant collection covers 3476 genes (65.25%) from the 5327 genes in the STm 14028s genome, not including the 1124 genes (21.10%) that code for essential genes (e.g., tRNA, rRNA) (Porwollik et al., 2014). All bacterial strains, including the isolate STm SL1344 MB282 (Hoiseth and Stocker, 1981) were maintained in glycerol stock at −80°C and streaked on Low-Salt Luria-Broth (LSLB) agar plates at the time of use. Cultures grown on solid medium were kept stationary and incubated at 28°C until colonies were formed (∼24 h). Cultures grown in liquid LSLB medium were incubated at 28°C with rotational shaking (225 rpm). LSLB broth was prepared using 10 g Tryptone (VWR, J859-500G), 5 g Yeast Extract (BD, 212750), and 5 g NaCl (VWR, X190-1KG) per liter of water. LSLB Agar was prepared similarly with the addition of 15 g/L of Bacteriological Agar (VWR, J637-1KG). Medium was supplemented with either 60 μg/mL kanamycin or 20 μg/mL chloramphenicol to grow the STm mutant strains.

Bacterial cells of *P. syringae* pv. *tomato* (Pst) DC3000 or its non-polar mutant *hrc*C^–^ (Hauck et al., 2003), were grown in liquid LSLB medium at 28°C for all experiments. Cells were streaked on solid medium from frozen glycerol stocks for inoculum preparation. Medium was supplemented with rifampin (100 μg/mL) to grow Pst strains.

### Plant Material and Growth Conditions

Commercial iceberg lettuce (*L. sativa* L. var *crispa* cv. Salinas) seeds were a gift from the Michelmore lab at the University of California, Davis. Seeds were germinated for 2 days on damp paper towels at 20°C with a 12-h daylight cycle at 100 μE photosynthetic active radiation and 65 ± 5% air relative humidity (RH). Seedlings were transplanted to dampened Peat Pellets (Jiffy, 70000116) and incubated for 3 weeks with a 12-h daylight cycle at 200 μE photosynthetic active radiation. Conditions for temperature and air RH were 19 ± 1°C and 75 ± 4% RH during the day and 18 ± 1°C and 92 ± 2% RH at night. 3-week old plants were either used in some assays or transplanted into 8 cm^2^ pots (Kord Products, Toronto, SQA3500) for later use. Pots contained SunGro Professional Growing Mix supplemented with 20 mL of fertilizer solution (Peters^®^ Excel pH Low 19-11-21 at a concentration of 14.8 g/L) and treated with Gnatrol WDG (Outlaw Hydroponics, AJ-WYC7-RK9U) (1 g/L). Transplanted plants were used at 5 weeks of age and kept in a controlled environment chamber for the duration of the experiments.

Seeds of *Arabidopsis thaliana* (L. Heyhn.) wild type ecotype Columbia (Col-0, ABRC stock CS60000) were sown in a 1:1:1 (v:v:v) mixture of growing medium (Redi-earth plug and seedling mix, Sun Gro), fine vermiculite, and perlite. Plants were grown in controlled environmental chambers at 22 ± 2°C, 60 ± 10% RH, and a 12-h photoperiod under light intensity of 100 μE photosynthetic active radiation. For all experiments, 4–5-week old plants were used.

### Stomatal Circadian Movement Evaluation

Stomatal aperture widths were measured as described by Montano and Melotto (2017). Briefly, sections from four leaves from 3-week-old lettuce plants were excised and the abaxial surface was imaged with a Nikon Eclipse Ni-U upright microscope (Nikon Corporations, Shinagawaku, Tokyo, Japan). Images were recorded every 2 h during the daylight time (0–12 h) using the Nikon NIS Elements Imaging Software Version 4.13.04 and stomatal aperture was determined as mean (*n* = 80 ± standard error) of eight biological replicates (i.e., four different leaves and the experiment performed twice). Ten stomata were imaged per leaf. Significant differences among the means were tested by ANOVA with *post hoc* Tukey HSD test (*p* < 0.05).

### MGD Mutant Library Screen

Multi-gene deletion mutants were screened for their ability to stimulate stomatal re-opening at 4 h post-inoculation (hpi) as compared to the parental strain STm 14028s. Bacterial liquid cultures were allowed to grow to an OD_600_ of 0.8–1.0. Cells were harvested by centrifugation (1360 × *g*, 20°C) and resuspended in water to generate an inoculum concentration of 1 × 10^8^ CFU/mL (0.2 OD_600_). Each bacterial inoculum (3 mL) was dispensed in one well of a 12-well plate and two sections of 3-week old lettuce leaves were floated abaxial side down on the inoculum as reported by Chitrakar and Melotto (2010). Images of abaxial surface stomata were captured at 4 hpi as previously described (Montano and Melotto, 2017). Mean stomatal aperture width (*n* = 20) and standard error (SE) were calculated from two independent leaves and compared by Student’s *t*-test.

Owing that a large number of mutants were not able to re-open the stomatal pore in this first screening (177 MGD strains), we functionally annotated the missing genes in these strains based on the description of the mutants described in Supplementary Table S2 from Porwollik et al. (2014) as another criterion for selecting mutants for further investigations. Functional annotation of genes and operons were conducted by BLAST searches using NCBI^1^ and KEGG (Kanehisa et al., 2017) databases. We then selected 51 mutants to be re-tested as described above, but this time using three biological replicates. Further selection of ten MGD mutants was based on their consistent inability to open the stomatal pore and predicted function of mutated genes. A workflow of the functional genetic screen is depicted in Supplementary Figure S1.

### Genotyping of Mutant Strains

Genome mutation in the ten selected MGD strains was confirmed by genome sequencing and PCR analyses. Genomic DNA (gDNA) was extracted from MGD strains and the wild type STm 14028s using DNeasy UltraClean Microbial Kit (Qiagen, Germantown, MD, United States) according to the manufacturer’s instructions. High quality gDNA was submitted for library preparation and shotgun sequencing by the UC Davis DNA Technology Core using a MiSeq platform (PE300; Illumina, San Diego, CA, United States). Reads were assembled into scaffolds using A5-miseq for KBase^2^ and aligned to published *Salmonella* genomes, STm 14028s (Jarvik et al., 2010) (NCBI accession number NC_016856.1) and STm LT2 (McClelland et al., 2001) (NCBI accession number NC_003197.2), using the NCBI megablast tool (Tatusova and Madden, 1999) with default parameters. All MGD strain sequences and our STm 14028s isolate aligned to the published STm 14028s with >99% identity (*E*-values = 0.0). The deleted region of each MGD mutant strain was identified and functionally annotated. Missing functional units were inferred through analysis of the functional annotation of the STm 14028s and STm LT2 regions available at NCBI. The STm 14028s annotation is current as of February 2017 and was created using the NCBI Prokaryotic Genome Annotation Pipeline (Tatusova et al., 2016). The LT2 annotation is current as of September 2017 and was created using the programs GLIMMER (Delcher et al., 1999) and GeneMark (Borodovsky and McIninch, 1993).

To confirm the *in silico* prediction of the deletion site, gDNA from the MGD strains was amplified with primers flanking each predicted deleted region, while the corresponding wild type STm 14028s genomic regions were amplified using a forward primer flanking the deletion start site and a reverse primer located within the deleted region, except for Mut9, for which both forward and reverse primers were located within the deleted region. PCR reactions were carried out using 200 ng gDNA, 100 nM of each primer (Supplementary Table S4), and GoTaq Green Master Mix (Promega, Madison, WI, United States) diluted with nuclease free water to a final volume of 50 μl. Thermocycler (Bio-Rad, Hercules, CA, United States) conditions included one initial 2 min period of 95°C and 30 cycles of 95°C for 30 s, 53°C for 30 s, and 73°C for 2 min, followed by a final 5 min period of 72°C. PCR products and Bio-Rad EZ Load^TM^ 1 kb Molecular Ruler #1708355 (Bio-Rad) were visualized using SYBR Safe DNA gel stain (Invitrogen, Carlsbad, CA, United States) in 1% agarose gel and purified with Promega Wizard SV Gel and PCR Clean-Up system (Promega, Madison, WI, United States). Purified DNA fragments were submitted for Sanger sequencing at the UC Davis Facility^3^ to confirm the exact start and end sites of the deletion.

### Stomatal Movement in Response to Selected Mutants in Mature Lettuce Plants

Five-mL of LSLB bacterial cultures were grown in 14.0 mL culture tubes on a rotary shaker (150 rpm) to an OD_600_ of 0.8–1.0. Cells were harvested by centrifugation (1360 × *g*, 20°C) and resuspended in water to an OD_600_ of 0.002 (1 × 10^6^ CFU/mL). Inoculum was infiltrated with a needleless syringe into leaves of 5-week-old lettuce plants that were kept under the same environmental conditions used for plant growth. Stomatal bioassay was conducted as previously described (Montano and Melotto, 2017) at 2 and 4 hpi. Mean stomatal aperture widths (*n* = 120; 40/biological replicate) from three independent leaves and SE were calculated. The difference between the means (mutant versus wild type) was compared by Student’s *t*-test to determine statistical significance.

### Bacterial Motility Assay

Swimming motility was assessed by analyzing movement within low-percentage agar (VWR, United States) as previously described (O’May and Tufenkji, 2011). Briefly, bacterial cultures were grown in LSLB liquid medium at 160 rpm to an OD_600_ of 0.8–1.0. Cells were harvested via centrifugation (1360 × *g*, 20°C) for 20 min and resuspended in sterile water to an OD_600_ of 0.2 (1 × 10^8^ CFU/mL). One microliter of this bacterial culture was inoculated within the swim agar medium (LSLB with 0.3% agar) followed by incubation for 24 h at 30°C. Turbidity resulting from bacterial migration from the inoculation point through the low-percentage agar was assessed. Qualitative results were recorded by imaging the culture plates. The assay was repeated three times with three replicates each time.

To assess bacterial swarming motility based on the protocol described by O’May and Tufenkji (2011), freshly streaked LSLB agar plates grown overnight was used to obtain single colonies of a similar size and age. One single colony was used to inoculate on the surface of a swarm agar medium (LSLB with 0.6% agar). Medium plates were incubated at 95 ± 5% humidity at 30°C for 24 h. Motility was assessed by measuring the distance of the swarm from the point of inoculation. Mean swarm distance (*n* = 8 ± SE) was calculated from eight independent plates assessed in two separate assays and statistical significance between the means (mutant versus wild type) was assessed using Student’s *t-*test.

### Bacterial Persistence in the Leaf Apoplast

Bacterial cultures were grown in LSLB liquid medium at 160 rpm to an OD_600_ of 0.8–1.0. Cells were harvested by centrifugation (1360 × *g*, 20°C) and resuspended in water to an OD_600_ of 0.002 (1 × 10^6^ CFU/mL). This inoculum was infiltrated into 5-week-old lettuce leaves with a needleless syringe as previously described (Katagiri et al., 2002). Apoplastic bacterial titer was evaluated by serial dilution plating technique as previously described (Jacob et al., 2017) at 0, 3, 7, 14, and 21 days post-inoculation (dpi). Briefly, the inoculated leaf was detached from the plant, surface-sterilized (1 min 2% sodium hypochlorite solution, 1 min 70% ethanol, 1 min sterile deionized water), followed by mechanical maceration of leaf disks with known area and plating. Data represents the mean of four technical replicates and three biological replicates (i.e., three leaves) per strain per time point, repeated twice at different days. Mean (*n* = 24 ± SE) bacterial population size was calculated and statistical significance was evaluated using the Student’s *t*-test comparing each mutant to STm 14028s at each time point.

### Callose Deposition Assay

To assess the strength of apoplastic defenses of lettuce cultivar Salinas against *Salmonella* strains and compare to that of the Arabidopsis/*Pseudomonas* system, a callose deposition assay was performed as previously described (Hauck et al., 2003; Nguyen et al., 2010). Briefly, attached whole leaves of 5-week old plants were infiltrated with either water (mock treatment) or 1 × 10^8^ CFU/mL of bacterium inoculum using a needless syringe as described by Katagiri et al. (2002). After 24 h, leaves were harvested and chlorophyll was cleared by immersing the leaves into 95% ethanol and kept at 37°C until chlorophyll was removed completely. Ethanol was replaced whenever necessary. Cleared leaves were rinsed consecutively in 70% ethanol and water followed by a 1-h incubation with 150 mM K_2_HPO_4_ containing 0.01% aniline blue. For microscopy, leaves were mounted on slides using 50% glycerol and imaged under a Nikon Eclipse Ni-U upright microscope equipped with DAPI filter. Damaged areas, mid vein, and leaf edges were avoided for imaging to prevent false positive results. Light intensity settings were set to 1350–2047 and LUTs were set to 850–2047. Analysis of the images was performed using the NIS Elements Imaging Software Version 4.13.04 (Nikon). Six images from each of four biological replicates (i.e., four leaves) per treatment were recorded and analyzed and the assay was repeated three times. Mean (*n* = 12 ± SE) callose deposits/mm^2^ was calculated and statistical significance was determined with a one-way ANOVA with *post hoc* Tukey HSD (*p* < 0.05) using XLSTAT version 19.4.

### Bacterial Growth in Apoplastic Wash Fluid

Apoplastic wash fluid (AWF) was extracted from 5-week-old lettuce leaves, omitting the cotyledons, using an infiltration-centrifugation method as previously described (O’Leary et al., 2014). To ensure that plant cellular contamination did not occur during extraction, AWF was evaluated for cellular contaminants using the Sigma-Aldrich^®^ Glucose-6-Phosphate Dehydrogenase Assay Kit (Sigma-Aldrich, MAK015-1KT). None of the AWF used exhibited detectable levels of G6PDH (data not shown). AWF was saved in aliquots to limit freeze-thaw cycles and stored at −20°C and filter sterilized at the time of use.

Bacterial cultures were grown in LSLB liquid medium on an orbital shaker to an OD_600_ of 0.8–0.1. Cells were harvested by centrifugation (1360 × *g*, 20°C) and resuspended in water to an OD_600_ of 0.2 (1 × 10^8^ CFU/mL). An aliquot of this inoculum was added to each medium (water, LSLB, or AWF) to achieve an initial bacterial culture concentration of 5 × 10^6^ CFU/mL in a 96-well plate format. Growth curves were obtained by growing cultures stationary, except for a 30-s rotation prior to each OD_600_ reading using a BioTek EPOCH 2 Microplate Spectrophotometer (BioTek, Winooski, VT, United States). OD_600_ readings were obtained every 30 min throughout a 24-h period and blanks (sterile media) were included as a control. This experiment was performed three times with three technical replicates each time. Mean OD_600_ (*n* = 9 ± SE) for each time point of the growth curve was calculated after subtracting the mean blank value and subsequently converted to bacterial cell number per mL of culture.

Growth rates (generations/hour) in the log-phase of growth were determined using the formula *N*_0_*x* 2^*n*^ = *N*_f_ where *N*_*0*_ is the number of bacteria at the first time point of interest, *N*_f_ is the number of bacteria at the final time point of interest, and *n* is the number of generations. The formula was rearranged to $l\,o\,g_{2}\,\frac{N_{\text{f}}}{N_{0}}=n$ to calculate *n* and the number of generations *n* was divided by the time to obtain the number of generations per hour as previously described (Todar, 2012).

## Results

### *Salmonella* Mutant Screening for the Inability to Re-open Lettuce Stomata

**FIGURE 1:**
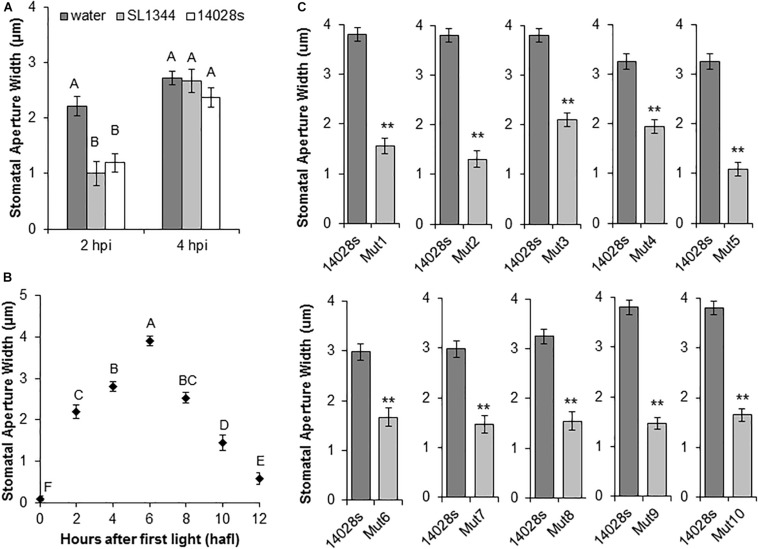
*Salmonella enterica* serovar Typhimurium (STm) mutant screening analysis. **(A)** STm 14028s and STm SL1344 induce similar stomatal reactions in lettuce leaves at 2 and 4 h post-inoculation (hpi). Leaf sections were floated on bacterial suspensions (1 × 10^8^ CFU/mL). Results are shown as the mean (*n* = 20) ± standard error (SE) calculated from two independent leaves. Different letters above the bars indicate statistically significant differences among the means (ANOVA with *post hoc* Tukey HSD test; *p* < 0.05). **(B)** Circadian rhythm of stomatal movement on non-inoculated lettuce leaves. Mean stomatal aperture widths (*n* = 80 ± SE) of eight biological replicates (i.e., four different leaves and the experiment performed twice). Ten stomata were imaged per leaf. Different letters above the bars indicate statistically significant differences among the means (ANOVA with *post hoc* Tukey HSD test; *p* < 0.05). **(C)** Stomatal response to selected bacterial mutants. Lettuce leaf sections were floated on bacterial inoculum (1 × 10^8^ CFU/mL) and stomatal aperture widths were measured at 4 hpi. Results are shown as mean stomatal aperture width (*n* = 120; 40/biological replicate) from three independent leaves and SE. Statistical difference between the means (STm 14028s vs. mutant) was determined using Student’s *t*-test (**p < 0.01).

We utilized a collection of MGD bacterial mutants derived from STm strain 14028s (Porwollik et al., 2014). We first confirmed that this strain induces a similar stomatal response to that of STm strain SL1344 (Roy et al., 2013). We evaluated changes in the stomatal aperture width in leaves of young lettuce plants by floating leaf pieces onto bacterial inoculum as previously reported (Chitrakar and Melotto, 2010). Both STm strains induced an initial stomatal closure at 2 h post inoculation (hpi) followed by re-opening at 4 hpi (Figure 1A), suggesting that the MGD library could be useful to identify STm genomic regions required for successful stomatal re-opening at 4 hpi. Second, to ensure that lack of re-opening was due to deletion of genes required for stomatal re-opening by STm 14028s rather than temporal factors, the circadian movement of lettuce stomata was determined. This analysis indicated that the stomatal aperture was widest at 6 h after first light (hafl) (Figure 1B). We therefore, chose to start the stomatal bioassay at 2 hafl to ensure that the 4 hpi time point corresponded to a time with maximum expected stomatal aperture width.

A primary screen of 303 MGD strains with a single biological replicate indicated that 177 mutants were unable to re-open stomata, suggesting a high rate of false-positives. Thus, we functionally annotated the predicted deleted genes in these 177 mutants (Porwollik et al., 2014). Considering the current knowledge of STm epiphytic behavior (Kroupitski et al., 2009), we reasoned that genes involved in secretion, perception of environmental signals, signaling, and regulatory functions could be involved in opening of the stomatal pore. Thus, we selected 51 MGD mutants based on their functional annotation for re-testing with at least three biological replicates. The primary functional units missing in these 51 mutants are described in Supplementary Table S2. From this confirmation screen, only eight mutants (named Mut1/2/4/5/6/7/8/10) were unable to re-open lettuce stomata consistently (Supplementary Table S2) and they were selected for further characterization. Furthermore, previous results indicated that mutants for the *Salmonella* Pathogenicity Island 1 and 2 (SPI-1 and SPI-2) were unable to open lettuce stomatal pores (S. Sela, unpublished data). Thus, we also analyzed two MGD strains from our collection (Mut3 and Mut9) that have a predicted deletion of these regions in addition to a few adjacent genes (Figure 1C and Table 1).

**TABLE 1:** Functional unit annotation for the ten MGD mutant strains selected for further characterization.

Mutant	Plate-well position	Deleted nucleotides	Deletion size (bp)	Deleted gene loci (ID)
Mut1	K_77/78_C08	4538998–4572514	33,516	STM14_RS22490 to STM14_RS22630
Mut2	K_77/78_F03	3618835–3626190	7,355	STM14_RS18330 to STM14_RS18370
Mut3	C_03_H10	2998648–3042149	43,501	STM14_RS15195 to STM14_RS15425
Mut4	C_01_H4	2451061–2455149	4,088	STM14_RS12670 to STM14_RS12690
Mut5	C_01_G2	2016000–2046442	30,442	STM14_RS10460 to STM14_RS10615
Mut6	C_01_F12	1948041–1981245	33,204	STM14_RS10090 to STM14_RS10285
Mut7	C_01_E9	1572754–1583690	10,936	STM14_RS08285 to STM14_RS08335
Mut8	C_01_E6	1534872–1548754	13,882	STM14_RS08105 to STM14_RS08175
Mut9	K_71/72_E4	1462743–1511389	48,646	STM14_RS07705 to STM14_RS07965
Mut10	C_02_G4	1051514–1057804	6,290	STM14_RS05455 to STM14_RS05480

*The plate-well position corresponds to the original MGD collection reported by Porwollik et al. (2014). Full annotation of deleted genes is listed in Supplementary Table S3.*

**FIGURE 2:**
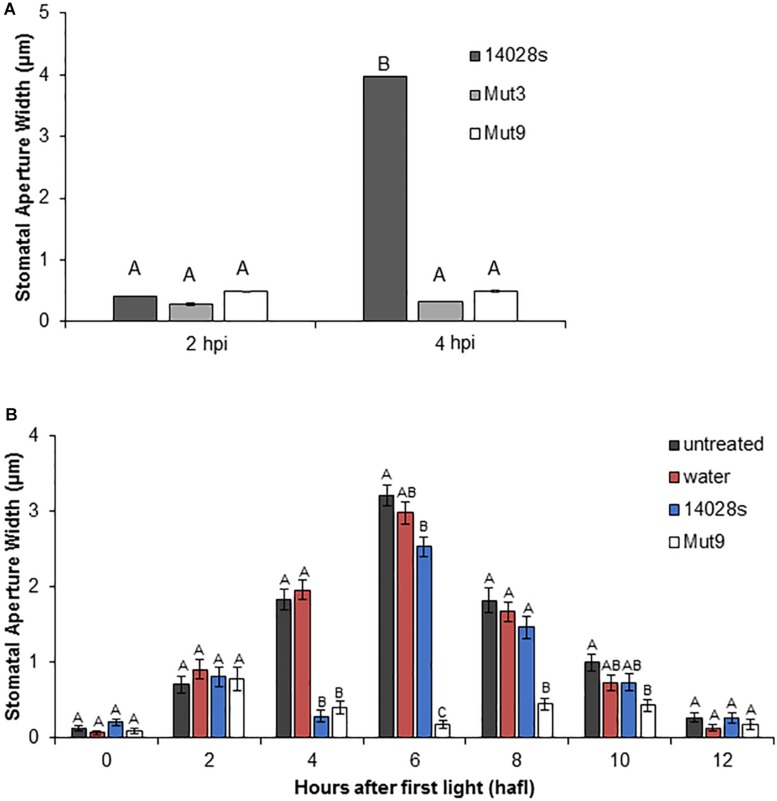
Stomatal response to Mut3 and Mut9. **(A)** Bacterial inoculum (2 × 10^6^ CFU/mL) of *Salmonella* mutants and 14028s was syringe-infiltrated into 5-week-old *L. sativa* cv. Salinas leaves. Inoculation of leaves was performed 2 h after first light (hafl). Results are shown as the mean (*n* = 120 ± SE) stomatal aperture width in three independent leaves measured at 2 and 4 hpi. Note that error bars are too small to appear in the graph. Different letters above adjacent bars indicate statistically significant differences among the means within each time point as determined by two-way ANOVA with *post hoc* Tukey HSD test (*p* < 0.01). **(B)** Long-term impact of inoculation with 14028s or Mut9 on the stomatal circadian rhythm on intact *L. sativa* cv. Salinas plants from 0 hafl until darkness (12 hafl). Leaves either remained untreated or were infiltrated with water or a bacterial inoculum (2 × 10^6^ CFU/mL) after 2 hafl. Mean stomatal aperture width (*n* = 80 ± SE) of eight biological replicates (i.e., four different leaves and the experiment performed twice). Ten stomata were imaged per leaf. Statistical significance of samples within each time point was determined using a one-way ANOVA with *post hoc* Tukey HSD test (*p* < 0.01). Different letters above adjacent bars indicate statistically significant differences among the means.

To confirm that the lack of stomatal re-opening using leaf pieces floating on bacterial inoculum (i.e., surface inoculation of detached leaves) was a reproducible response that can also be observed in leaves still attached to the plant, we designed a stomatal bioassay that included infiltration of mature lettuce leaves with STm 14028s, Mut3, or Mut9 bacterium suspensions. In this assay, bacteria are placed in the leaf apoplast, including the sub-stomatal chamber, where they can be in contact with the guard cells. All three strains induced a strong stomatal closure at 2 hpi (Figure 2A), similar to observations made using surface inoculation of mature, whole plants (Roy et al., 2013; Montano and Melotto, 2017). Furthermore, the wild type strain STm 14028s, but not the mutant strains, induced stomatal re-opening at 4 hpi (Figure 2A), suggesting that this response is robust. To rule out the possibility that the infiltration procedure induced an unpredictable stomatal movement, we assessed the circadian stomatal movement in untreated lettuce leaves as well as leaves infiltrated with water (mock control), STm 14028s, or Mut9. Mock-treated and untreated leaves showed an almost identical movement pattern, stomata of Mut9-infiltrated leaves remained closed throughout the daylight period, and STm 14028s-infiltrated leaves showed a transient reduction in stomatal aperture width at 2 hpi that corresponded to 4 hafl (Figure 2B).

### Genotypic and Phenotypic Characterization of Selected Mutants

**FIGURE 3:**
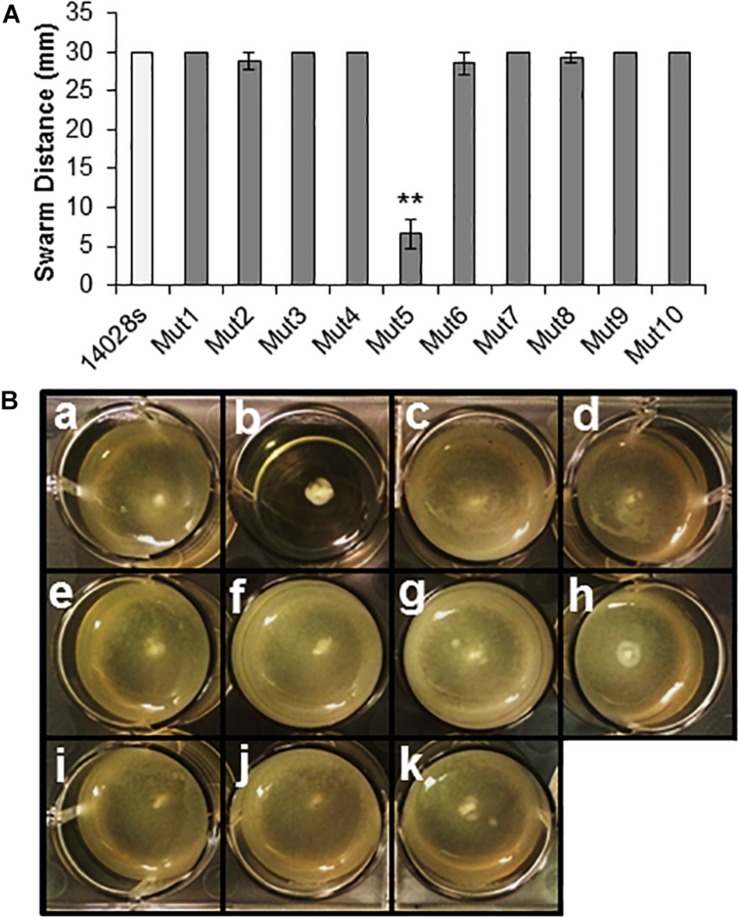
Motility assays using the ten selected MGD mutants. **(A)** Swarming motility was assayed on soft agar by inoculating a freshly grown bacterial colony on top of the agar and incubating the culture under high RH (95 ± 5%) for 24 h. Single colony inocula were of similar size and age. Results are shown as the mean (*n* = 8) from eight independent plates assessed in two separate assays. Note that some error bars are too small to appear on the graph scale. Statistical analysis (STm 14028s vs. mutant) was performed using a Student’s *t*-test (**p < 0.01). **(B)** Swimming motility assay on soft agar inoculated with 1 uL of bacterial culture (1 × 10^8^ CFU/mL) within the agar and observed at 24 hpi. Diffuse turbidity indicates movement of cells from the point of inoculation (center of the well) and confirmed swimming capability in STm 14028s (a), Mut4 (c), Mut2 (d), Mut9 (e), Mut8 (f), Mut7 (g), Mut1 (h), Mut3 (i), Mut10 (j), and Mut6 (k). Lack of movement from the point of inoculation was observed for mutant Mut5 (b). The assay was performed three times with three replicates per assay, with consistent results.

Upon completion of this screening procedure, the genome position of the deleted region for each mutant was identified at the nucleotide level by whole genome sequencing of the mutant strains. This procedure, which was readily available in a time- and cost-effective matter, allowed us to predict the genotype and the functional units missing in each mutant using the available STm 14028s and LT2 genome annotations (Table 1 and Supplementary Table S1) (McClelland et al., 2001; Jarvik et al., 2010). The genomic regions deleted in all ten selected mutants were also confirmed by PCR (Supplementary Figure S2). Furthermore, each mutant, except Mut5, was able to swim and swarm (Figure 3), confirming the predicted genotype of Mut5 is missing genes involved in flagellar biosynthesis and chemotaxis (Supplementary Table S3). Movement and chemotaxis have previously been associated with STm SL1344 internalization through the stomatal pore (Kroupitski et al., 2009). Thus, the identification of Mut5 during the *Salmonella* genetic screening validates our procedure, which identified known and novel features associated with bacterial epiphytic behavior. The 10 MGD strains were further tested for phenotypic traits required for colonization of leaves as described below.

### MGD Mutants Have Variable Apoplastic Persistence

As all selected mutants were unable to stimulate stomatal re-opening at 4 hpi (Figure 1C), we sought to determine whether each mutation also affected the population dynamics in the lettuce leaf apoplast. To characterize each mutant’s ability to survive within the apoplast, leaves were infiltrated with bacterial inoculum. This allowed for direct analysis of population titer changes due to apoplastic interactions and eliminated confounding factors, such as failure to survive on the leaf surface and/or lack of internalization through stomata. Apoplastic titers were followed over a 3-week period to capture the dynamics of population growth.

**FIGURE 4:**
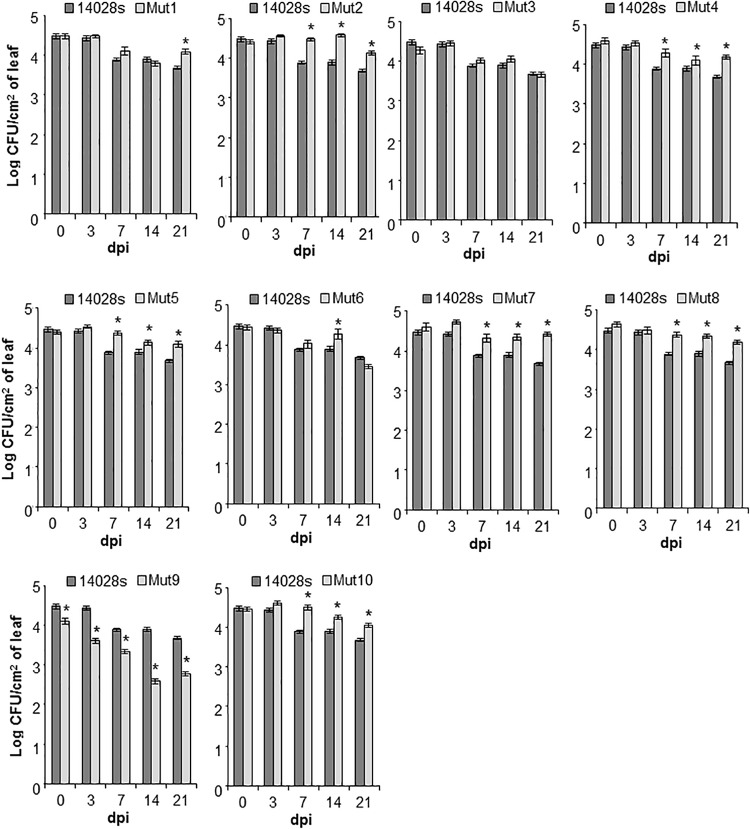
Bacterial persistence in the apoplast of lettuce. Leaves of intact *L. sativa* cv. Salinas plants were syringe-infiltrated with bacterial inoculum (1 × 10^6^ CFU/mL). Bacterial titers were followed over a 3-week period and enumerated at 0, 3, 7, 14, and 21 days post-inoculation (dpi) using a serial-dilution plating assay. Results are shown as mean (*n* = 24 ± SE) obtained from four technical replicates and three biological replicates (i.e., three leaves) repeated twice. The statistical significance between the means (STm 14028s vs. mutant at each time point) was determined using a Student’s *t*-test (*p < 0.05). Lack of a star on top of the bar indicates no statistical difference.

The wild type bacterium STm 14028s population declined significantly (*p* < 0.05) in lettuce leaves (from an average of 3 × 10^4^ CFU/cm^2^ at day 0 to an average of 4.7 × 10^3^ CFU/cm^2^ at day 21), while variable population titers were observed among the mutants (Figure 4). For instance, seven mutants, Mut1/2/4/5/7/8/10, had significantly (*p* < 0.05) greater population titers (between 0.5 and 1 log increase) than that of the wild type bacterium at 21 dpi, whereas Mut3 and Mut6 apoplastic persistence did not differ from that of the wild type at 21 dpi (Figure 4).

Interestingly, only Mut9 showed significantly (*p* < 0.05) impaired endophytic survival (Figure 4). This finding indicates that genes missing in this mutant, including the SPI-2 and the *suf*, *ynh*, *lpp*, and *ttr* operons (Supplementary Table S3), may be required for the bacterium to cope with or overcome plant defenses and/or the ability of the bacterium to obtain nutrients from the apoplastic environment necessary to maintain its population. To test for these possibilities, we performed a callose deposit assay and a bacterial growth rate assay using lettuce apoplastic wash fluid (AWF).

### STm 14028s Does Not Suppress Callose Deposition Through SPI2 or SPI1

**FIGURE 5:**
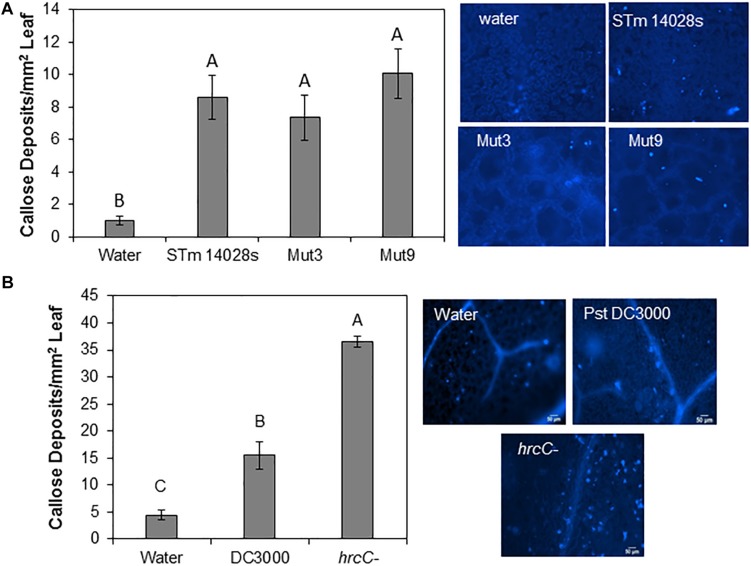
Callose deposition assay. 4–5-week-old lettuce **(A)** or Arabidopsis **(B)** plants were syringe-infiltrated with a 1 × 10^8^ CFU/mL bacterial inoculum (*S. enterica* or *P. syringae*) or water. Callose deposits were enumerated and bars represent the mean (*n* = 12 ± SE) number of deposits per mm^2^ of leaf obtained from four different leaves and the assay was repeated three times. Statistical significance among the means was determined by one-way ANOVA with *post hoc* Tukey HSD (*p* < 0.05). Different letters above the bars indicate statistically significant differences. Images on the right show typical deposit patterns after each treatment.

Callose deposition is a hallmark plant defense response that is induced upon biotic stress (Hauck et al., 2003). Thus, we determined the average number of callose deposits in lettuce leaves inoculated with STm 14028s, Mut3, and Mut9. We observed that all three bacteria induced similar numbers of callose deposits that were significantly higher than those seen in the water control (Figure 5A). Because all three STm strains induced a relatively low number of callose deposits (average of 7–10 deposits/mm^2^), we also inoculated Arabidopsis with the virulent phytopathogen Pst DC3000 for comparison with this well-established system. As previously reported by Hauck et al. (2003), Pst DC3000 did not induce strong callose deposition on its Arabidopsis plant host (approximately 15 deposits/mm^2^), although callose deposit frequency was significantly higher than in the water control (Figure 5B). However, the Pst DC3000 type-three secretion system (TTSS) mutant (*hrc*C^–^) induced 2.5 times more callose deposits than the wild type Pst DC3000 in Arabidopsis leaves (Figure 5B).

Altogether, our findings suggest that STm 14028s can induce a weak defense in lettuce leaves, similar to that of Pst DC3000 in Arabidopsis leaves. A major function of the SPI genomic region is to assemble the TTSS apparatus and encode effector proteins that could potentially suppress plant defenses. However, we observed that, unlike in the Arabidopsis-Pst DC3000 pathosystem where the TTSS is involved in suppressing plant immune response such as callose (Hauck et al., 2003), the SPI-1 and SPI-2 regions of STm 14028s are not involved in this process in the lettuce system.

### Mut9 Growth Is Impaired in Lettuce AWF

**FIGURE 6:**
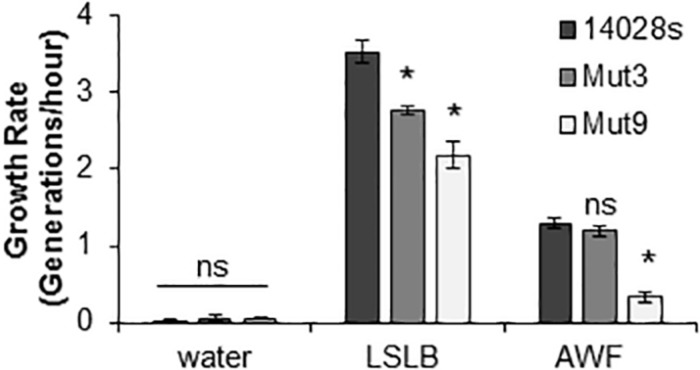
Bacterial growth in different media. Generation times in the log-phase of growth was determined for STm 14028s, Mut3, and Mut9. Results are shown as mean growth rate (*n* = 9 ± SE). This experiment was performed three times with three technical replicated each time. Statistical significance between the means (STm 14028s vs. mutant in each medium) was determined using a Student’s *t-*test (*p < 0.05). ns = no statistical difference, LSLB, low-salt Luria-Bertani; AWF, apoplastic wash fluid.

Growth rates (generation/hour) of STm 14028s, Mut3, and Mut9 in AWF and LSLB were determined during the log-phase of bacterial growth. Water was used to identify growth rates under a no-nutrient condition. As expected, there was minimal bacterial growth in water (Figure 6), indicating that residual nutrients in the inoculum were not transferred to LSLB or AWF to enhance growth. In an attempt to correlate the ability of the bacterium to survive within the apoplast (Figure 4) with the ability to utilize apoplastic nutrients for growth, we included in this analysis Mut3 that contains a deletion of SPI-1 and adjacent genes (Supplementary Table S3) and shows apoplastic persistence similar to the wild type STm 14028s (Figure 4). When grown on LSLB, both Mut3 and Mut9 had statistically significant (*p* < 0.05) lower growth rates than STm 14028s. Mut3, Mut9, and STm 14028s had growth rates of 2.78, 2.18, and 3.53 generations/hour, respectively (Figure 6). When grown in lettuce AWF, Mut3 and STm 14028s had similar growth rates, while the Mut9 growth rate was significantly lower (Figure 6). This finding suggests that the STm’s ability to persist in the apoplast may be linked to nutrient acquisition or the overall bacterial fitness in this niche that is dependent on yet-to-be determined gene(s) and operon(s) deleted in Mut9.

## Discussion

The importance of foodborne illness caused by contamination of produce by *Salmonella* spp. and the prevalence of contamination associated with leafy greens (DeWaal et al., 2008) led us to investigate the molecular mechanisms allowing *Salmonella* spp. to use this alternate host for survival. As apoplastic populations of human pathogenic bacteria in lettuce are a potential risk for foodborne illnesses due to persistence from production to consumption, we directed our focus on the bacterial internalization into leaves through stomata and endophytic survival. *S. enterica* internalization of leaves can occur through the stomatal pore (Kroupitski et al., 2009; Roy et al., 2013).

We were able to identify ten regions in the STm 14028s genome that may directly or indirectly contribute to the bacterium’s ability to open the stomatal pore facilitating its entry into the apoplast. Although it is not obvious which genes in those regions are specifically responsible for the observed phenotype on the leaf surface, the major metabolic functions of these regions are associated with sensing the environment, bacterium chemotaxis and movement, membrane transporters, and biosynthesis of surface appendices (Supplementary Table S3). Previously, these functions have been found to be associated with epiphytic fitness of bacterial phytopathogens (reviewed by Melotto and Kunkel, 2013 and Pfeilmeier et al., 2016). Furthermore, Kroupitski et al. (2009) observed that STm SL1344 aggregates near open stomata and uses chemotaxis and motility for internalization through lettuce stomata. Additionally, darkness prevents STm SL1344’s ability to re-open the stomatal pore (Roy et al., 2013) and internalization into the leaves possibly due to the lack of chemoattractant leaching through closed stomata (Kroupitski et al., 2009). These findings suggest that close proximity to stomata may be required for *Salmonella* to induce opening of the pore. Therefore, STm invasion of the apoplast may be a consequence of a combined behavior of the bacterium on the phylloplane that can be modulated by plant-derived cues and, with this study, we have defined key genomic regions involved in this complex process.

Not all the genomic regions required for initiation of the leaf colonization (i.e., epiphytic behavior and tissue penetration) are essential for continuing bacterial survival as an endophyte (Figure 4). For instance, genes deleted from Mut3 (encoding SPI-1) and Mut6 [encoding unspecified membrane proteins, the PhoP/Q two-component system, SopE2 (an effector involved in mammalian infection) (Gong et al., 2009), phage genes, a transcriptional repressor (KdgR), and some unspecified transporters] do not contribute to endophytic survival. Thus, these regions missing in Mut3/6 are potential targets for disrupting leaf surface colonization, but not endophytic persistence. This observation is not entirely surprising as the phylloplane and the apoplast environments are unique and they pose different challenges for bacterial survival in these niches. STm seems to have metabolic plasticity for adaptation to varying conditions in the leaf. For instance, STm SL1344 can shift its metabolism to utilize nutrients available in decaying lettuce and cilantro leaves (Goudeau et al., 2013) and STm 14028s uses distinct metabolism strategies to colonize tomatoes and animal infection (de Moraes et al., 2017).

We also observed that seven regions of the STm 14028s genome have opposite effects on the different phases of colonization. Mut1/2/4/5/7/8/10 seem to lack the ability to promote penetration into the leaf (Figure 1C), but they show better fitness than that of the wild type strain in the apoplast (Figure 4). One hypothesis is that the increased bacterial population titers are due to lack of energy expenditure for maintaining large genomic segments that are not essential for survival as an endophyte, so that the excess energy can be spent on survival. However, this indirect effect of the deletion may not be valid for Mut4/10, where only small genomic regions are missing (Supplementary Table S3). Alternatively, these regions might encode for proteins that negatively affect bacterial survival in leaves. This interesting observation is worth future investigation.

Intriguingly, we found that genes deleted in Mut9 are important for re-opening the stomatal pore and successful endophytic survival. This deletion includes SPI-2 that functions in the production of the TTSS-2 apparatus, effectors, and a two-component regulatory system of this island (Coombes et al., 2004), which are important for the virulence of STm in animal systems (Waterman and Holden, 2003). The contribution of the TTSS-2 apparatus and effectors to the bacterium’s ability to colonize the phyllosphere has been studied in several laboratories and it is largely dependent on the plant species analyzed (reviewed by Garcia and Hirt, 2014 and Melotto et al., 2014). Nonetheless, so far there is no evidence for the ability of STm to inject TTSS effectors inside plant cells (Chalupowicz et al., 2018). Furthermore, the STm 14028s *ssaV*-structural mutant, that cannot form the TTSS-2 apparatus (Vishwakarma et al., 2014), survives in the lettuce cv. Romit 936 to the same extent as the wild type bacterium after surface inoculation (Chalupowicz et al., 2018). Our data also support the notion that the TTSS-2 is not involved in STm ability to induce or subvert defenses, such as callose deposition in lettuce cv. Salinas (Figure 5). While studies in other plant systems have suggested that TTSS and encoded effectors may contribute to bacterial survival in the plant environment (Schikora et al., 2011; Shirron and Yaron, 2011) or in some cases are detrimental for bacterial colonization of plant tissues (Iniguez et al., 2005), it has become evident that the TTSS-2 within the SPI-2 region is not relevant in the STm 14028s-lettuce leaf interaction.

It is important to note that SPI-2 is a genomic segment of roughly 40 kb with 42 open reading frames arranged into 17 operons (Supplementary Table S3) (Hensel, 2000). It is present in all pathogenic serovars and strains of *S. enterica*, but only partially present in species of a more distant common ancestor, such as *S. bongori* (Hensel, 2000). Besides encoding structural and regulatory components of the TTSS-2 (Coombes et al., 2004), SPI2 also carries genes coding for a tetrathionate reductase complex, a cysteine desulfurase enzyme complex, membrane transport proteins, murein transpeptidases, as well as genes with still uncharacterized functions (NCBI Resource Coordinators, 2017). Thus, it is possible that genes and operons, other than the ones associated with TTSS-2, may have a function in the bacterium colonization of the lettuce leaf.

To date, it has not been demonstrated whether STm 14028s can access and utilize nutrients from the apoplast of intact lettuce leaves. Although nutrients in the apoplast might be limiting (Lindow and Brandl, 2003), it has been hypothesized that *Salmonella* may scavenge nutrients to persist in the plant environment (Teplitski and de Moraes, 2018) and/or adjust its metabolism to synthesize compounds that are not readily available at the colonization site. For instance, a mutant screen analysis indicated that STm 14028s requires genes for biosynthesis of nucleotides, lipopolysaccharide, and amino acids during colonization of tomato fruits (de Moraes et al., 2017). Moreover, plants might secrete antimicrobial compounds into the apoplast as a plant defense mechanism, imposing a stressful condition to the microbial invader (reviewed by Doehlemann and Hemetsberger, 2013). Therefore, considering that subversion of plant defenses is not a function of the TTSS-2 in the apoplast of lettuce (Figure 5), it is possible that the Mut9 population reduces 20 fold over 21 days (Figure 4) due to its inability to obtain nutrients from this niche and/or to cope with plant defenses. Although Mut9 shows reduced growth on lettuce leaf AWF (Figure 6), additional experimentation is required to distinguish between these two possibilities. It is tempting to speculate, however, that the tetrathionate reductase gene cluster (*ttrRSBCA*) within SPI-2 or the sulfur mobilization (SUF) operon deleted in Mut9 (Supplementary Table S3) might be involved in this process. Particular to the ttr operon, TtrAB forms the enzyme complex, TtrC anchors the enzyme to the membrane, whereas TtrS and TtrR are the sensor kinase and DNA-binding response regulator, respectively (James et al., 2013). The reduction of tetrathionate by this membrane-localized enzyme is part of the *Salmonella*’s anaerobic respiration (Hensel et al., 1999). Intriguingly, the use of tetrathionate as an electron acceptor during propanediol and ethanolamine utilization by the bacterium (Price-Carter et al., 2001) has been suggested to occur in macerated leaf tissue (Goudeau et al., 2013). A significant number of genes involved in the PDU (propanediol utilization), EUT (ethanolamine), and cobalamin pathways as well as the *ttrC* gene are upregulated in STm SL1344 when co-inoculated with the soft rot pathogen *Dickeya dadantii* onto cilantro and lettuce leaf cuts (Goudeau et al., 2013). Altogether, these findings suggest that these biochemical pathways may occur in both soft rot contaminated and healthy leaves.

Considering that the encounter of the plant with a pathogenic bacterium triggers molecular action and reaction in both organisms overtime, it is not surprising that multiple regions of the STm 14028s genome may be required for lettuce leaf colonization. For instance, Goudeau et al. (2013) reported that 718 (16.4%) genes of the STm SL1344 genome were transcriptionally regulated upon exposure to degrading lettuce cell wall. In any case, further studies using single-gene mutants are still required to identify the specific genes and functions within each MGD mutant that are involved in the interaction between STm 14028s and lettuce cultivar Salinas.

## Data Availability Statement

The datasets generated for this study are available upon request to the corresponding author.

## Author Contributions

MMe conceived the research. JM and MMe designed the research and wrote the manuscript. JM, GR, JT, and SP performed the experiments. JM, GR, and MMe analyzed the data. SS, MMc, and MMe provided materials and discussed the project in detail. All authors read and approved the manuscript.

## Conflict of Interest

The authors declare that the research was conducted in the absence of any commercial or financial relationships that could be construed as a potential conflict of interest.

## Footnotes

1 https://blast.ncbi.nlm.nih.gov/Blast.cgi

2 http://kbase.us

3 http://dnaseq.ucdavis.edu/

## Supplementary Material

FIGURE S1 Multi-gene deletion (MGD) mutant strain screen design. **(A)** 3-week old *L. sativa* cv. Salinas plants grown in Peat Pellets. Leaves from plants of this stage were used in leaf float stomatal assays. **(B)** Leaf sections of the *L. sativa* cv. Salinas floated, abaxial side down, on bacterial inoculum (1 × 10^8^ CFU/mL) or water control were used for stomatal assays according to Montano and Melotto (2017). **(C)** Schematic of three-layer screen to identify ten mutants for detailed characterization.

FIGURE S2 Genotype confirmation of selected MGD mutants. **(A)** Diagram showing primer locations. Shaded areas represent wild type sequence that is present in both the wild type and the mutant strains. White areas represent sequence that is unique to either the wild type or the mutant. WTFP, Wild Type Forward Primer; WTRP, Wild Type Reverse Primer; MFP, Mutant Forward Primer; MRP, Mutant Reverse Primer. **(B)** Agarose gel electrophoresis of PCR reactions containing the indicated gDNA and primer sets. The far left lanes contain the molecular weight marker with band sizes indicated in Kb. No amplification was expected in the samples loaded onto Lanes 2 and 4.

TABLE S1 List of 303 MGD mutant strains screened for the inability to re-open the stomatal pore. Plate-well numbers are based on previously published data (Porwollik et al., 2014).

TABLE S2 Functional unit overview for 51 MGD mutant strains selected for a confirmation screen. Plate-well number and predicted mutation site are based on previously published data (Porwollik et al., 2014). The ability of each strain to re-open stomata (yes, no, or partial) was determined by comparisons with the wild type STm 14028s (yes) and Mut9 (no) phenotypes using Student’s *t*-test (*p* < 0.01). MGD mutant strains classified as “partial” for the stomatal re-opening ability indicates that the phenotype of those strains was intermediate between STm 14028s and Mut9. Functional annotation was inferred by BLAST analysis using the NCBI database. Strains in bold letters were chosen for further analysis.

TABLE S3 Genes deleted in each MGD mutant strain and their corresponding annotation based on the published genomes of the STm strains 14028s and LT2.

TABLE S4 Primers sequences and target genomic regions to validate the mutation site of each MGD strain.
