# Supplementary figures and images for: Salmonella enterica Serovar Typhimurium 14028s Genomic Regions Required for Colonization of Lettuce Leaves
Source: Front Microbiol. 2020 Jan 24;11:6. doi: 10.3389/fmicb.2020.00006 (PMC6993584; doi:10.3389/fmicb.2020.00006)

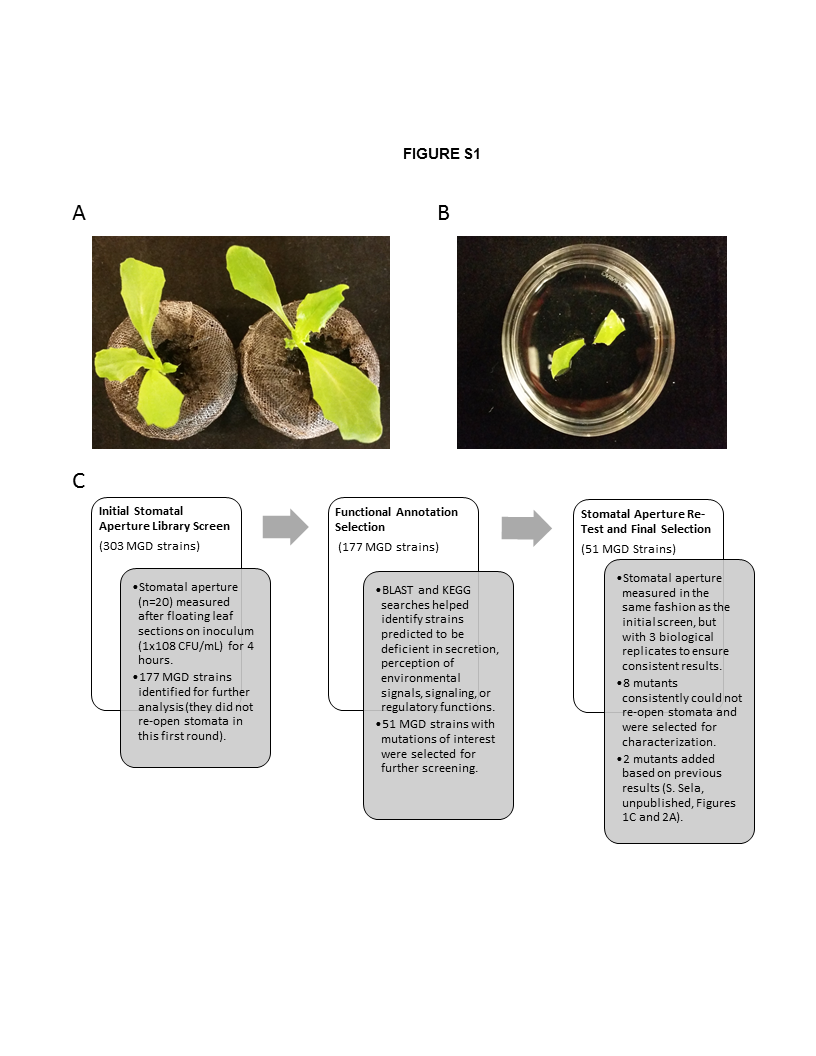

Supplement: FIGURE S1 — Multi-gene deletion (MGD) mutant strain screen design. (A) 3-week old L. sativa cv. Salinas plants grown in Peat Pellets. Leaves from plants of this stage were used in leaf float stomatal assays. (B) Leaf sections of the L. sativa cv. Salinas floated, abaxial side down, on bacterial inoculum (1 × 10^8^ CFU/mL) or water control were used for stomatal assays according to Montano and Melotto (2017). (C) Schematic of three-layer screen to identify ten mutants for detailed characterization. [file Image_1.TIF]

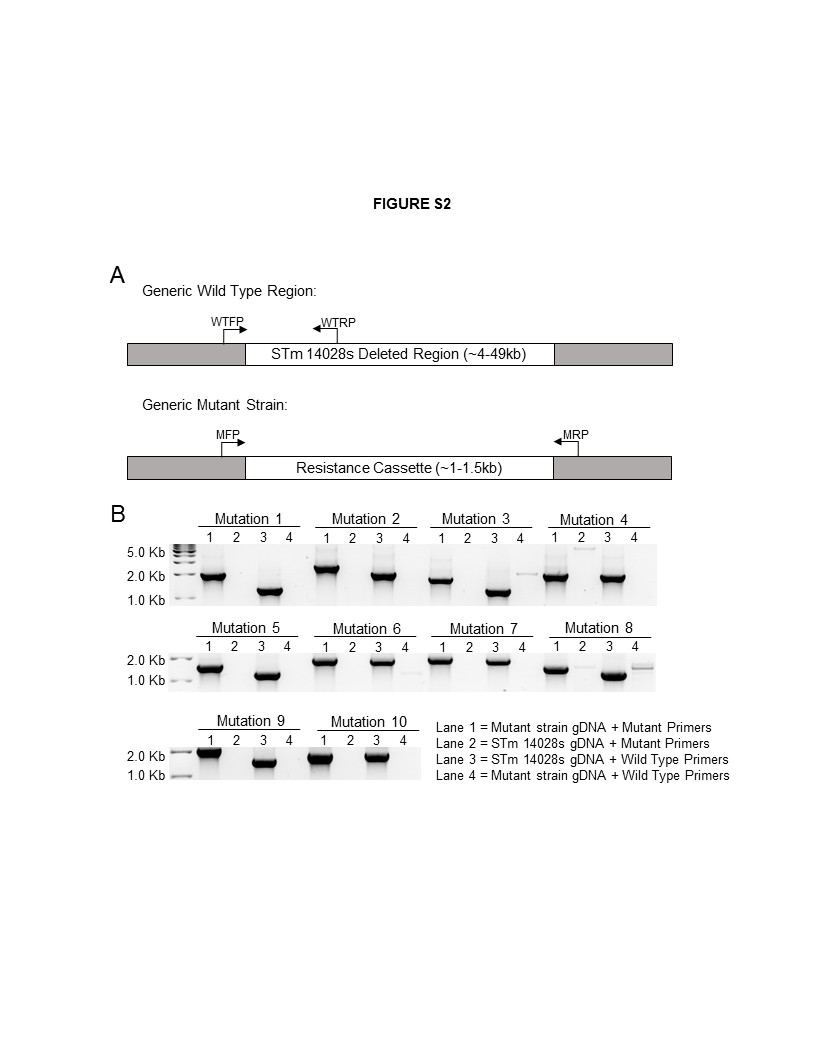

Supplement: FIGURE S2 — Genotype confirmation of selected MGD mutants. (A) Diagram showing primer locations. Shaded areas represent wild type sequence that is present in both the wild type and the mutant strains. White areas represent sequence that is unique to either the wild type or the mutant. WTFP, Wild Type Forward Primer; WTRP, Wild Type Reverse Primer; MFP, Mutant Forward Primer; MRP, Mutant Reverse Primer. (B) Agarose gel electrophoresis of PCR reactions containing the indicated gDNA and primer sets. The far left lanes contain the molecular weight marker with band sizes indicated in Kb. No amplification was expected in the samples loaded onto Lanes 2 and 4. [file Image_2.TIF]
